# The Korean National Codes Against Cancer: background of their establishment and the revision process

**DOI:** 10.4178/epih.e2025027

**Published:** 2025-05-14

**Authors:** Yoonjoo Choi, Jin-Kyoung Oh, Ayoung Byeon, Byungmi Kim

**Affiliations:** 1 Division of Cancer Prevention, National Cancer Control Institute, National Cancer Center, Goyang, Korea; 2 Department of Public Health & AI, Graduate School of Cancer Science and Policy, National Cancer Center, Goyang, Korea

**Keywords:** Cancer, Primary prevention, Recommendation, Guideline, Evidence-based process, Consensus

## Abstract

The Korean National Codes Against Cancer provide guidelines for cancer prevention. The inaugural edition was published in 2006, followed by a major revision in 2016. This study aimed to describe the historical context, scientific rationale, and revision process of these guidelines. With rising cancer incidence in the early 2000s, the Korean government recognized the need for national cancer prevention guidelines, prompting the National Cancer Center to initiate their formulation. The Division of Cancer Prevention reviewed global literature on cancer trends and Korea-specific studies on cancer risk factors. The final set of 10 recommendations comprising the Korean National Codes Against Cancer was approved by the National Cancer Control Committee after achieving expert consensus on cancer prevention. The finalized guidelines are firmly grounded in scientific evidence. The 10 current recommendations include: (1) no smoking and avoidance of secondhand smoke; (2) consuming sufficient fruits and vegetables as part of a balanced diet; (3) reducing salt intake and avoiding burnt or charred foods; (4) limiting alcohol consumption; (5) engaging in regular physical activity (at least 30 minutes a day, 5 days a week); (6) maintaining a healthy body weight; (7) receiving immunization against hepatitis B virus and human papillomavirus; (8) practicing safe sex by maintaining a single sexual partner and using condoms; (9) following health and safety guidelines to avoid exposure to occupational carcinogens; and (10) undergoing regular cancer screening. This study detailed the sources and procedures involved in formulating and revising the Korean National Codes Against Cancer.

## GRAPHICAL ABSTRACT

[Fig f2-epih-47-e2025027]

## Key Message

The Korean Code Against Cancer comprises a set of recommendations intended to prevent cancer. These guidelines were initially established and published in 2006 and subsequently revised in 2016 to incorporate accumulated research findings and evolving societal factors. The Secretariat of the Division of Cancer Prevention at the National Cancer Center, in collaboration with expert working groups from various fields, systematically reviewed relevant studies, gathered opinions, and reached consensus to ensure that the guidelines were evidence-based. The National Cancer Control Committee approved the currently published 10 codes for cancer prevention.

## INTRODUCTION

Cancer has been the leading cause of death in Korea since 1983, the year when cause-of-death statistics were first published. In response, the Ministry of Health and Welfare (MOHW) introduced the inaugural National Cancer Control Plan in 1995. By the early 2000s, Korea’s cancer management infrastructure had expanded considerably, characterized by the establishment of the Cancer Policy Division, creation of the National Cancer Center, and enactment of the Cancer Control Act in 2003. In 2005, the Division of Cancer Prevention was specifically established to enhance prevention initiatives.

During this period, evidence increasingly indicated that many cancer cases were associated with preventable risk factors. The World Health Organization (WHO) estimated that 30% to 50% of cancer cases could be prevented, while the remaining cases could be managed effectively through early detection and appropriate treatment [1,2]. A Korean study analyzing cancer data from 2009 estimated that 33.8% of new cancer cases and 45.2% of cancer-related deaths were attributable to modifiable risk factors, including smoking (11.9% for incidence and 22.8% for mortality), chronic infections (20.1 and 23.6%), alcohol consumption (1.8 and 1.8%), obesity (1.8 and 1.3%), physical inactivity (0.7 and 0.3%), and reproductive and hormonal factors (1.6 and 0.9%). These findings underscored the significant impact of lifestyle and environmental factors on cancer incidence, highlighting the necessity for targeted prevention strategies [3].

Additionally, Giddens [4]’ concept of the “investing state,” promoted by the United Kingdom’s Tony Blair administration in the early 2000s, influenced participatory governance in Korea. This concept emphasized investment in human capital and the promotion of healthy lifestyles. At that time, however, Korea’s healthcare system primarily prioritized treatment over prevention [5]. Recognizing the importance of prevention, the MOHW incorporated “cancer prevention through risk factor management” as a key strategy in its Second National Cancer Control Plan of 2006. The initial step of this strategy was enhancing public awareness through the dissemination of cancer prevention guidelines, culminating in the establishment of the Korean National Codes Against Cancer.

The Korean National Codes Against Cancer were developed and officially released following a rigorous systematic review of scientific evidence, extensive expert consultations, evaluations from multiple relevant institutions, and approval from the National Cancer Control Committee. However, comprehensive documentation outlining the foundational evidence and procedural framework of these guidelines remains to be established.

Therefore, this study aimed to systematically examine the development process and historical context of these guidelines, providing a valuable reference for future revisions and facilitating the systematic advancement of cancer prevention strategies.

### Development process of the Korean National Codes Against Cancer in 2006

The Division of Cancer Prevention, acting as the secretariat, reviewed national cancer data and existing literature on cancer risk factors in Korea to formulate evidence-based guidelines. Expert advisory working groups (WGs) convened multiple meetings beginning in May 2006.

Around the same period, the WHO issued its own cancer prevention recommendations [1], and the European Union formulated the “European Code Against Cancer” [6]. Similarly, the United Kingdom established the “Healthy Living: Guidelines to Help You Avoid Cancer,” and Japan introduced its “12 Guidelines for Cancer Prevention” [7]. These international trends prompted the secretariat and WGs to develop 15 preliminary guidelines focusing on smoking, alcohol consumption, diet, physical activity, vaccinations, and screening practices based on Korea-specific cancer data.

The draft was subsequently reviewed by 43 organizations, including the MOHW, relevant governmental agencies, academic societies, and hospital associations, to ensure social acceptability. After incorporating feedback from these stakeholders, 10 guidelines were finalized.

The following principles guided the selection and wording of the finalized guidelines: (1) Include recommendations related to clear health risks based on the precautionary principle; (2) Prioritize items with scientifically validated evidence of health risks and preventive benefits; (3) Exclude recommendations with insufficient or inconsistent evidence; (4) Use clear and straightforward language to facilitate public understanding, consolidating overlapping items into 10 concise guidelines.

**Figure 1.**
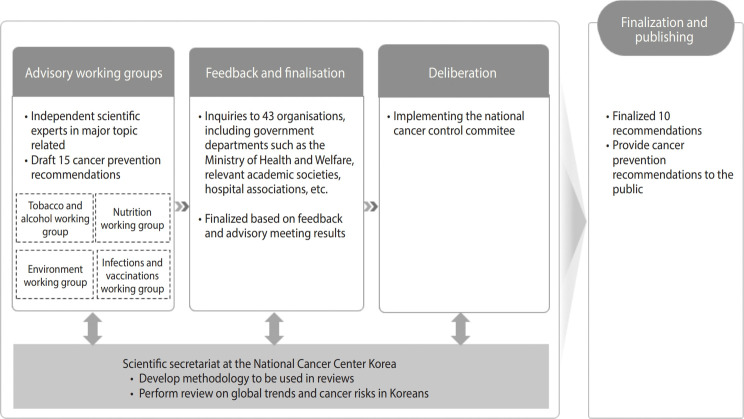
The scientific development process of the Korean National Codes Against Cancer.

The finalized guidelines, approved by the National Cancer Control Committee, were officially announced by the MOHW in October 2006 (Figure 1). These guidelines covered recommendations related to smoking, diet (2 items), alcohol consumption, physical activity, obesity, infections (2 items), occupational exposures, and early detection (Table 1).

#### First revision of the Korean National Codes Against Cancer in 2016

By the mid-2010s, changing social conditions, lifestyle trends, and emerging scientific evidence highlighted the need to revise the cancer prevention guidelines. Therefore, the National Cancer Center initiated the first revision in line with changes observed in global guidelines.

This revision primarily focused on recommendations related to alcohol consumption and vaccination. Initially, the 2004 European Code Against Cancer recommended moderate alcohol consumption, citing cardiovascular health benefits [6]. Subsequent research, however, demonstrated that even minimal alcohol intake could increase cancer risk, indicating no identifiable “safe” threshold [8]. Alcohol had been classified as a group 1 carcinogen by the International Agency for Research on Cancer (IARC) since 1987; this classification was reinforced by additional evidence in 2010 [9]. The Global Burden of Disease Study identified alcohol as the seventh leading global risk factor for mortality and disability-adjusted life years [10], advocating complete abstinence to reduce health risks. Furthermore, a meta-analysis found that daily consumption of ≤12 g of alcohol increased risks for oral and pharyngeal cancer by 17%, esophageal cancer by 30%, breast cancer by 5%, liver cancer by 8%, and colorectal cancer by 7% [11]. Based on this evidence, the European Code Against Cancer revised its guidelines in 2014 to recommend complete abstinence from alcohol for cancer prevention [12].

International organizations revised their alcohol guidelines based on well-established evidence indicating: (1) alcohol consumption has a direct carcinogenic mechanism; (2) a dose-response relationship exists, with cancer risk increasing as alcohol intake increases; (3) this relationship is consistent across all alcohol types; (4) abstaining from alcohol reduces cancer risk; and (5) even minimal consumption (one drink or less daily) increases cancer risk. Collectively, these findings suggest no safe level of alcohol consumption regarding cancer risk.

Responding to this global consensus, the National Cancer Center revised its guideline from “limit to no more than two drinks daily” to simply “limiting alcohol consumption.”

Additionally, the vaccination guideline was updated. Initially, only hepatitis B vaccination was included. After the WHO compiled evidence on the safety and efficacy of the human papillomavirus (HPV) vaccine for cervical cancer prevention, policies were implemented to broaden HPV vaccination coverage. Cervical cancer, primarily caused by HPV infection, can be prevented effectively (>94%) through vaccination administered before the onset of sexual activity. Consequently, HPV vaccination was integrated into Korea’s National Immunization Program (NIP). Since June 2016, HPV vaccinations have been provided free of charge to 12-year-old girls (sixth-grade students). Including HPV vaccination in the NIP represented a significant policy update during the revision of cancer prevention guidelines.

Despite the preventive efficacy of these measures, financial feasibility remains crucial when developing universally applicable cancer prevention guidelines. Economic constraints associated with implementation can hinder adherence, particularly in low-income populations, thereby reducing overall public health impact. These financial considerations further supported the rationale behind revising the cancer prevention guidelines.

The initial revision followed the original 2006 development process, involving extensive expert consultations and contributions from relevant institutions and academic societies. The updated guidelines were officially announced by the MOHW in March 2016, commemorating Cancer Prevention Day.

The original and revised versions of the Korean National Codes Against Cancer are presented in Table 1.

## SOURCES OF EVIDENCE FOR THE GUIDELINES

The secretariat conducted a comprehensive literature review to formulate and revise the Korean National Codes Against Cancer. Evidence was systematically reviewed to develop and refine the 10 recommendations, and relevant sources were provided for associated questions and answers. The finalized guidelines included recommendations related to smoking, diet (2 items), alcohol consumption, physical activity, healthy weight, infection-related risks (2 items), occupational carcinogen exposure, and early cancer screening.

### No smoking and avoiding secondhand smoke

Smoking and secondhand smoke are well-established cancer risk factors [13]. Nicotine, tar, formaldehyde, and benzene found in tobacco smoke significantly increase the risk of cancers, including leukemia and cancers of the lung, mouth, stomach, esophagus, larynx, kidney, bladder, cervix, and pancreas. Specifically, smoking elevates lung cancer risk by approximately 20-fold [14,15]. According to the IARC, smoking is classified as a group 1 carcinogen [13].

Secondhand smoke, generated by burning cigarettes or exhaled by smokers, also elevates cancer risk, particularly for cancers of the lung, breast, and larynx [13]. The 2006 Surgeon General’s Report unequivocally stated that there is no safe level of secondhand smoke exposure [14], highlighting that exposure increases lung cancer risk among non-smokers by 20-30%. Similar risks for exposed non-smokers have been reported by the WHO and the United States Environmental Protection Agency [13]. Consequently, many countries have implemented stricter public smoking bans, recognizing the particular vulnerability of children and older adults. The WHO and the Centers for Disease Control and Prevention recommend smoking cessation and minimizing secondhand smoke exposure as global strategies for cancer prevention. The Korean guidelines have thus emphasized the critical importance of this recommendation.

### Consuming a sufficient amount of fruits and vegetables and a balanced diet

The abundant antioxidants, fiber, vitamins, and minerals present in fruits and vegetables play a significant role in the prevention of cancer and cardiovascular disease [16]. The WHO recommends increasing the intake of these foods to mitigate disease risk [17].

Studies suggest that sufficient fruit and vegetable consumption can decrease overall cancer incidence by approximately 5-12%. Fruit intake specifically reduces the risk of lung, bladder, mouth, pharyngeal, laryngeal, esophageal, stomach, and colorectal cancers, whereas vegetable intake contributes to the prevention of esophageal, breast, lung, stomach, and colorectal cancers [18]. The WHO, World Cancer Research Fund, and American Institute for Cancer Research have systematically compiled evidence linking diet and cancer risk, providing the foundation for cancer prevention programs and policies worldwide [19]. The WHO specifically advises consuming at least 400 g of fruits and vegetables daily to achieve adequate fiber intake [20]. Similarly, the American Cancer Society emphasizes the importance of a balanced diet rich in fruits and vegetables, rather than narrowly focusing on individual nutrients [21]. Aligned with international recommendations and evidence, the Korean Codes Against Cancer identify sufficient consumption of fruits and vegetables and maintaining a balanced diet as essential cancer prevention strategies.

### Reducing salt intake and avoiding burnt or charred foods

A diet high in salt or charred foods increases the risk of stomach and other cancers [22]. Salted and pickled foods may irritate the gastric mucosa, leading to inflammation and consequently elevating stomach cancer risk [23]. In Korea, where stomach cancer rates are notably high, salty food consumption is particularly prevalent. The WHO and IARC recommend consuming less than 5 g of salt daily due to its established link to cancer [23]. National health guidelines in Japan, Europe, and the United States similarly advocate low-salt diets for cancer prevention [24].

Charred or burnt foods contain carcinogens, including polycyclic aromatic hydrocarbons and heterocyclic amines, which form when proteins or fats are cooked at high temperatures [25]. These substances can induce genetic mutations, thereby increasing cancer risk [26]. Foods commonly prepared through barbecuing, grilling, or frying have been associated with increased risks of colon and stomach cancers [27]. Studies have proposed avoiding high-temperature cooking methods to prevent food charring. Therefore, Korea’s cancer prevention guidelines explicitly recommend reducing salt intake and avoiding the consumption of charred foods.

### Limiting alcohol consumption

In 2006, the Korean National Codes Against Cancer recommended limiting alcohol consumption to no more than 2 drinks daily, defining 1 drink as containing 12 g of pure alcohol. This recommendation aligned with international standards, including guidelines from the WHO and the National Institutes of Health, which defined risky drinking as consuming 3 or more drinks daily or excessive weekly intake [28]. Moderate alcohol consumption was subsequently thought to offer protective benefits against cardiovascular disease and diabetes [29].

However, later research identified significant methodological flaws in earlier studies, particularly the “sick quitter” bias, which had distorted conclusions about alcohol’s health effects [30]. Current evidence strongly links alcohol consumption to cancers of the mouth, pharynx, larynx, esophagus, liver, colorectum, and breast. Excessive alcohol intake increases upper gastrointestinal cancer risk by up to 9.2-fold and liver cancer risk by as much as 35-fold [8]. Acetaldehyde, a toxic byproduct produced during alcohol metabolism, damages DNA and promotes carcinogenesis. The IARC classified alcohol as a group 1 carcinogen, attributing approximately 3% of global cancer deaths to alcohol-related cancers [1].

Emerging evidence confirms that even moderate alcohol consumption increases cancer risk, with no identifiable safe intake threshold. Global burden-of-disease studies demonstrate a clear dose-response relationship, revealing that minimal alcohol intake heightens cancer risks, particularly for oral, pharyngeal, laryngeal, and esophageal cancers [10]. A meta-analysis encompassing 112 studies corroborated the elevated cancer risk associated with even low levels of alcohol consumption [11]. Consequently, the fourth European Code Against Cancer, published in 2014, recommended complete abstinence from alcohol as a cancer prevention measure [12]. Reflecting this global consensus, Korea revised its guideline from “limit to no more than two drinks daily” to “limiting alcohol consumption,” underscoring the consensus regarding alcohol’s significant cancer risks.

### Engaging in regular physical activity (at least 30 minutes, 5 days a week)

Regular physical activity reduces cancer risk through multiple mechanisms, including decreased inflammation, improved immune function, and reduced cellular oxidative damage [31]. A review of approximately 170 epidemiological studies linked low physical activity with increased risks for colorectal, breast, prostate, endometrial, and lung cancers [31].

Physical activity can reduce colorectal cancer risk by approximately 20% to 50%, depending on the intensity and duration of exercise. Women who regularly engage in vigorous exercise may experience breast cancer risk reductions of around 20-30% [32]. The WHO recommends at least 30 minutes of moderate exercise daily to reduce the risk of chronic diseases, such as cardiovascular disease and diabetes [19].

The 2005 Dietary Guidelines for Americans emphasize that high-intensity exercise, combined with healthy dietary practices and weight management, significantly reduces the risks of obesity, cancer, and cardiovascular diseases [33]. Based on these findings and international guidelines, the Korean Codes Against Cancer explicitly include regular physical activity as a major recommendation for cancer prevention.

### Maintaining a healthy body weight

Obesity, primarily caused by overeating, substantially increases the risk of chronic diseases, including cancer. Studies have linked obesity and physical inactivity with approximately 25-30% of major cancers, notably colorectal, breast, endometrial, kidney, and esophageal cancers [34]. A 2013 report from the National Cancer Center of Korea estimated that overweight and obesity accounted for approximately 2.8% of new cancer cases in Korea, with strong associations particularly noted with colorectal, breast, and endometrial cancers [3].

The 2005 Dietary Guidelines for Americans recommend calorie reduction and increased physical activity to maintain a healthy weight. According to the WHO’s Asia-Pacific classification, a healthy body weight corresponds to a body mass index between 18.5 kg/m2 and 23.0 kg/m².

Based on this evidence and international recommendations, maintaining a healthy body weight is prominently included in the Korean Codes Against Cancer as a central prevention strategy.

### Immunization against hepatitis B virus and human papillomavirus

Chronic hepatitis B virus (HBV) infection is strongly associated with liver cancer; a 2006 report indicated that 74.2% of hepatocellular carcinoma patients in Korea tested positive for hepatitis B surface antigen [35]. The HBV vaccine has demonstrated approximately 95% efficacy in preventing chronic HBV infection, making it the first recognized anticancer vaccine and a crucial preventive measure against liver cancer [35].

The Korean National Immunization guidelines recommend HBV vaccination at birth, followed by doses either at 1 month and 6 months or at 1 month, 2 months, and 18 months. Additionally, individuals at high-risk—including HBV-negative family members of HBV carriers, patients undergoing hemodialysis, injection drug users, healthcare workers, and those at risk for sexually transmitted infections—are advised to receive the vaccine [36].

Initially, the cancer prevention guidelines included only HBV vaccination. However, in the 2016 revision, HPV vaccination was incorporated following emerging evidence and updated policy guidelines. The WHO classifies HPV infection as a carcinogenic factor for cervical cancer, with HPV detected in over 99% of cervical cancer patients [37]. HPV types 16 and 18 account for approximately 70% of HPV-related cervical cancer cases. HPV vaccination effectively prevents these infections, reducing cervical cancer incidence by approximately 70%. In 2016, Korea integrated HPV vaccination into its NIP, recommending a 2-dose vaccination schedule for 12-year-old girls. This policy change led directly to the inclusion of HPV vaccination in the revised cancer prevention guidelines.

### Engaging in safe sex (having a single sexual partner and using condoms)

HPV infection, which is primarily transmitted through sexual intercourse, is a significant cause of cervical cancer and also contributes to oropharyngeal, anal, and cutaneous cancers. The risk of cervical cancer associated with HPV infection varies considerably, with relative risk values ranging from 3.6 to 573.4, depending on HPV type, geographical region, and study methodology [40]. However, persistent infection with high-risk HPV types—especially HPV-16 and HPV-18—remains the most critical risk factor for cervical cancer, although other factors such as smoking, oral contraceptive use, and coinfections also contribute [39].

In addition, HBV and hepatitis C virus (HCV), as well as human immunodeficiency virus (HIV), are associated with increased cancer risk. HBV and HCV infections are leading causes of hepatocellular carcinoma, while HIV infection weakens the immune system, significantly increasing the risk of cancers such as Kaposi sarcoma and various lymphomas.

Minimizing the number of sexual partners and consistently using barrier methods such as condoms are essential practices for preventing sexually transmitted infections and associated cancers.

### Following all health and safety instructions to prevent cancer-causing agent exposure in the workplace

Occupational exposure to carcinogens frequently occurs at young ages and often at high intensities, significantly increasing cancer risk among younger populations. These cancers typically affect tissues directly exposed to carcinogens, such as the skin and lungs, and vary depending on the specific carcinogen and the industrial processes involved.

A notable challenge in occupational carcinogen exposure is the prolonged latency period between exposure and disease onset, resulting in workers often being unaware of their carcinogen exposure until after extended periods of use. Therefore, adherence to occupational health and safety standards and consistent use of protective equipment are critical measures for mitigating cancer risk [13].

The WHO estimates that occupational exposures, particularly to substances such as asbestos and benzene, cause approximately 200,000 cancer-related deaths annually worldwide. It emphasizes strict adherence to safety protocols, the implementation of engineering safeguards, and workplace policies aimed at reducing exposure. Similarly, the European Agency for Safety and Health at Work advocates for comprehensive risk assessments, protective regulations, and detailed guidelines to mitigate exposure to industrial carcinogens [40]. In Korea, the Ministry of Employment and Labor’s Industrial Safety Bureau introduced the 11 Basic Health and Safety Principles in 2001, which are crucial elements of Korea’s workplace safety and cancer prevention strategies (Supplementary Material 1). These principles strongly emphasize minimizing occupational exposure to carcinogens as a critical component of cancer prevention efforts.

### Undergoing regular cancer screening

Cancer screening is essential for early cancer detection and reducing mortality. Screening identifies cancers at asymptomatic stages, significantly improving treatment success rates and patient survival. Some screening methods can detect and eliminate precancerous lesions, preventing their progression to invasive cancers. National cancer screening programs effectively target age groups at higher risk, serving both diagnostic and preventive roles.

The National Cancer Screening Program (NCSP) in Korea has demonstrated significant effectiveness in reducing cancer mortality. Jun et al. [41] reported that NCSP gastric cancer screening lowered gastric cancer mortality by 47%. Screening for colorectal cancer, especially through regular fecal occult blood tests and colonoscopy, has similarly reduced mortality. Additionally, liver cancer screening among high-risk individuals has notably improved early detection and decreased liver cancer mortality.

Endoscopic screening for gastric cancer in Japan has been linked to a 32% reduction in mortality. Annual colorectal cancer screening using colonoscopy or stool-based tests has similarly lowered mortality by 33% in the United States. Liver cancer screening has reduced liver cancer-specific mortality by approximately 37% in populations at elevated risk [42].

Although the benefit of mammography screening for women under 50 remains debated, mammography can reduce breast cancer mortality by approximately 35% [43]. Cervical cancer screening using Pap smears has successfully decreased cervical cancer incidence by approximately 3-4% per year and reduced cervical cancer mortality by more than 70% in the United States and Europe over the past 4 decades [44]. Due to their proven effectiveness, routine cancer screenings are universally recommended in cancer prevention guidelines.

## DISCUSSION

The formulation and dissemination of cancer prevention guidelines hold significant scientific and social importance. These guidelines are informed by recent research and statistical analyses, providing evidence-based information that enhances public awareness and understanding. Furthermore, such guidelines support the evaluation of cancer prevention initiatives and facilitate achieving global health objectives aligned with WHO frameworks [45]. By publicly promoting these guidelines, governments encourage healthier lifestyles, reduce cancer risks, and foster greater public engagement with health-related policies [46]. Public trust in official guidelines further strengthens community participation and improves the overall effectiveness of prevention strategies.

Since 2007, the Division of Cancer Prevention at the National Cancer Center has actively promoted cancer prevention efforts. The publication of these guidelines marked a crucial initial step in establishing Korea’s national cancer prevention policy, simultaneously influencing broader strategies for preventing chronic diseases.

The formulation and revision of the 10 recommendations within the Korean National Codes Against Cancer were guided by the following key principles: (1) including evidence-based recommendations; (2) prioritizing guidelines with scientifically validated health risks and preventive benefits; (3) excluding recommendations lacking robust evidence; and (4) enhancing readability and clarity by consolidating overlapping items into 10 final recommendations.

Following the release of these guidelines, biennial surveys have been conducted to evaluate public awareness and adherence. The latest survey in 2023 indicated that 80.3% of respondents recognized cancer as preventable. However, despite this high level of awareness, the actual implementation rate remained around 50%. The practice rate was notably lower among men and young adults in their 20s [47]. These findings provide valuable insights that inform ongoing cancer prevention policies and guide future revisions of the recommendations [46].

Important factors influencing these revisions include the increasing prevalence of inhaled tobacco use, the rising consumption of fast and processed foods, and recent research emphasizing the health benefits of physical activity. Given these developments, regular updates to the cancer prevention guidelines are necessary.

## CONCLUSION

Currently, the National Cancer Center is preparing a second revision following the initial revision conducted in 2016. The revision process involves systematically collecting scientific evidence, conducting thorough literature reviews, and consulting experts to produce practical, evidence-based guidelines that address the predominant cancers and risk factors specific to Korea. The revised guidelines will provide clear recommendations for cancer-preventive behaviors, contributing significantly to maintaining long-term public health at the population level.

### Ethics statement

This study did not involve human participants or the use of personally identifiable information. Therefore, it was confirmed by the Institutional Review Board (IRB) of the National Cancer Center Korea, that ethical approval was not required.

## Figures and Tables

**Figure f2-epih-47-e2025027:**
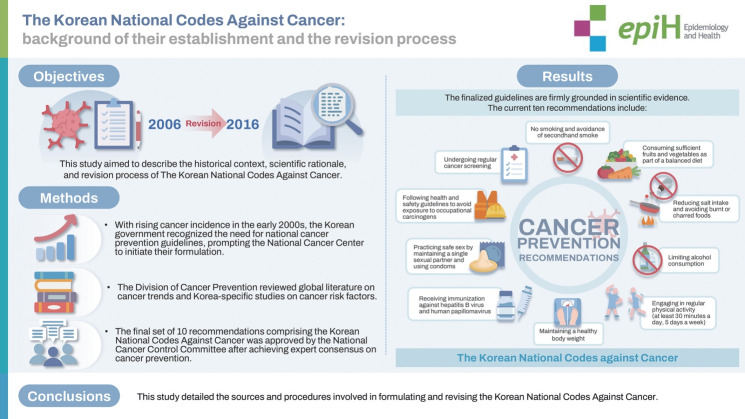


**Table 1.** Key changes from the 2006 to the 2016 Korean National Codes Against Cancer

Key changes	
Original guidelines in 2006	First revision in 2016
No smoking and avoiding secondhand smoke	No smoking and avoiding secondhand smoke
Consuming a sufficient amount of fruits and vegetables and a balanced diet	Consuming a sufficient amount of fruits and vegetables and a balanced diet
Reducing salt intake and avoiding burnt or charred foods	Reducing salt intake and avoiding burnt or charred foods
Limit alcohol to no more than two drinks a day	Limiting alcohol consumption
Engaging in regular physical activity (at least 30 minutes, 5 days a week)	Engaging in regular physical activity (at least 30 minutes, 5 days a week)
Maintaining a healthy body weight	Maintaining a healthy body weight
Immunization against hepatitis B virus	Immunization against hepatitis B virus and human papillomavirus
Engaging in safe sex (having a single sexual partner and using condoms)	Engaging in safe sex (having a single sexual partner and using condoms)
Following all health and safety instructions aimed at preventing exposure to cancer-causing agents in the workplace	Following all health and safety instructions aimed at preventing exposure to cancer-causing agents in the workplace
Undergoing regular cancer screening	Undergoing regular cancer screening

## Supplementary materials

Supplementary Material 1. The "11 Basic Health and Safety Principles"
