# Supplementary material for: The Korean National Codes Against Cancer: background of their establishment and the revision process
Source: Epidemiol Health. 2025 May 14;47:e2025027. doi: 10.4178/epih.e2025027 (PMC12433721; doi:10.4178/epih.e2025027)
Supplement: Supplementary Material 1. — The "11 Basic Health and Safety Principles" [file epih-47-e2025027-Supplementary-1.docx]

**Supplementary Material 1.** **The "11 Basic Health and Safety Principles":**

① Perform a safety check before beginning work and maintain neatness and orderliness during work.
② Ensure safe passageways at the worksite.
③ Provide and wear personal protective equipment.
④ Use insulating protective equipment when working with live electrical components.
⑤ When servicing or maintaining machines and facilities, use locking devices and warning signs.
⑥ Clearly place warning signs on hazardous chemicals.
⑦ Install protective devices on presses, shearers, pressure vessels, and circular saws.
⑧ Use safety handrails when working at heights, and securely cover all openings.
⑨ Install safety nets to prevent falls.
⑩ Keep combustible or explosive materials separate when performing welding operations.
⑪ Measure oxygen concentration levels before beginning work in enclosed or confined spaces.
